# Pharmacogenomics of platinum-based chemotherapy response in NSCLC: a genotyping study and a pooled analysis

**DOI:** 10.18632/oncotarget.9688

**Published:** 2016-05-29

**Authors:** Juan Chen, Zhan Wang, Ting Zou, Jiajia Cui, Jiye Yin, Wei Zheng, Wuzhong Jiang, Honghao Zhou, Zhaoqian Liu

**Affiliations:** ^1^ Department of Clinical Pharmacology, Xiangya Hospital, Central South University, Institute of Clinical Pharmacology, Central South University, Hunan Key Laboratory of Pharmacogenetics, Changsha, P. R. China; ^2^ Hunan Province Cooperation Innovation Center for Molecular Target New Drug Study, Hengyang, P. R. China; ^3^ Department of Oncology, Xiangya Hospital, Central South University, Changsha, P. R. China

**Keywords:** platinum, NSCLC, chemotherapy response, meta-analysis, polymorphism

## Abstract

Published data showed inconsistent results about associations of extensively studied polymorphisms with platinum-based chemotherapy response. Our study aimed to provide reliable conclusions of these associations by detecting genotypes of the SNPs in a larger sample size and summarizing a comprehensive pooled analysis. 13 SNPs in 8 genes were genotyped in 1024 NSCLC patients by SequenomMassARRAY. 39 published studies and our study were included in meta-analysis. Patients with GA or GG genotypes of XRCC1 G1196 had better response than AA genotype carriers (Genotyping study: OR = 0.72, 95%CI: 0.53-0.96, *P* = 0.028; Meta-analysis: OR = 0.74, 95%CI: 0.62-0.89, *P* = 0.001). Patients carrying CT or TT genotypes of XRCC1 C580T could be more sensitive to platinum-based chemotherapy compared to patients with CC genotype (OR = 0.54, 95%CI: 0.37-0.80, *P* = 0.002). CC genotype of XRCC3 C18067T carriers showed more resistance to platinum-based chemotherapy when compared to those with CT or TT genotypes (OR = 0.69, 95%CI: 0.52-0.91, *P* = 0.009). Our study indicated that XRCC1 G1196A/C580T and XRCC3 C18067T should be paid attention for personalized platinum-based chemotherapy in NSCLC patients.

## INTRODUCTION

Lung cancer is a serious health problem in the whole world for many decades with its highest mortality [1]. It is histologically consisted of small cell lung cancer (SCLC) and non-small cell lung cancer (NSCLC). NSCLC accounts for about 85% of the total cases. Platinum-based chemotherapy is the standard chemotherapy for first line treatment of NSCLC, especially for advance stage patients [2, 3]. However, the response to platinum-based chemotherapy is greatly variable among individuals, and drug resistance is easily occurred by intrinsic or acquired.

A number of studies suggested single nucleotide polymorphisms (SNPs) may affect chemotherapy response [4–9]. Most of the studies focused on the SNPs of genes in DNA repair pathways or transporters, such as ERCC1, XPD, XRCC1 and MDR1 [10–18]. However, inconsistent results came from different studies on the same issue. For example, Huang et al. and Zhao et al. reported that ERCC1 C8092A may be useful predictive markers for response to platinum-based chemotherapy [19, 20], but some other studies showed the contradictory results 21–23]. The same situations were also existed in MDR1 C3435T, XPD A2251C and other SNPs. Although several reviews and meta-analyses summarized the pharmacogenomics of platinum-based chemotherapy response in NSCLC patients [24], they still not reached the consistent conclusion. Moreover, the published meta-analyses were not comprehensive as they usually analyzed only one or several SNPs in their studies [25, 26].

In this study, we investigated the relationships between widely studied SNPs and platinum-based chemotherapy response in a larger NSCLC sample size. We also provided a comprehensive meta-analysis on pharmacogenomics of the platinum-based chemotherapy response. Understanding the genetic variants contributed to platinum-based chemotherapy will provide guide for individualized chemotherapy and benefit for NSCLC patients.

## MATERIALS AND METHODS

### Subjects

A total of 1024 NSCLC patients were enrolled in our genotyping study. They were from Shanghai Chest, Shanghai Zhongshan, or Shanghai Changhai Hospitals (Shanghai, China) from 2005 to 2010, and Affiliated Cancer Hospital or Xiangya Hospital of Central South University (Changsha, Hunan, China) from 2011 to 2015 (Table 1). The patients to be eligible for the study had to meet the following criteria: (1) histologically or cytologically confirmed NSCLC, and primary tumor in the lung; (2) Patients received platinum-based chemotherapy for at least two cycles, and had no surgery or radiotherapy before. Exclusion criteria included (1) pregnancy or lactation, (2) active infection, (3) symptomatic brain or leptomeningeal metastases, and (4) previous or concomitant other malignancies. Chemotherapy response was evaluated by Response Evaluation Criteria in Solid Tumors (RECIST) 1.1 [27]. Responders were consisted of complete responders (CR) and partial responders (PR) while non-responders including stable disease (SD) and progressive disease (PD). All subjects provided written informed consent, incompliance with the code of ethics of the World Medical Association (Declaration of Helsinki) before this study. The study protocol was approved by the ethics committee of Xiangya School of Medicine, Central South University (registration number: CTXY-110008-2). We applied for clinical admission to the Chinese Clinical Trial Registry (registration number: ChiCTR-RO-12002873).

**Table 1:** Clinical characteristics of responders and non-responders to platinum-based chemotherapy in NSCLC patients

Characteristics	Responders (%) (*n* = 237)	Non-responders (%) (*n* = 787)
Sex Male Female	174(73.4) 63(26.6)	550(69.9)) 237(30.1)
Age <55 ≥55	81(34.2) 156(63.8)	310(39.4) 477(60.6)
Smoking status Non-smoker Smoker	101(42.6) 136(57.4)	354(45.0) 433(55.0)
Stage IIIa-IIIb IV	92(38.8) 145(61.2)	241(30.6) 546(69.4)
Histology Squamous Adenocarcinoma	145(61.2) 92(38.8)	517(65.7) 270(34.3)
Chemotherapeutic regimen Platinum/gemcitabine Platinum/paclitaxel Platinum/vinorelbine	135(57.0) 55(23.2) 47(19.8)	357(45.4) 252(32.0) 178(22.6)

### SNP selecting, DNA extracting and genotyping

**Figure 1:**
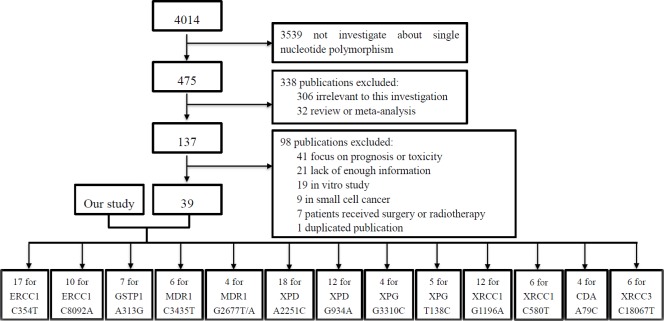
Flow chart of literature selection

By considering the meta-analysis of pharmacogenomics of platinum-based chemotherapy in NSCLC, we searched the publications which reported the associations of SNPs with platinum-based chemotherapy response of NSCLC in PubMed database, ISI Web of Knowledge and Cochrane Library. As shown in Figure 1, we found 13 SNPs were widely studied in different publications after systematic literature review. In order to get more reliable results by included more studies and with larger total sample size in the meta-analysis, we did genotyping study about these 13 SNPs in our samples. Information of the 13 SNPs was summarized in Table 2.

**Table 2:** Polymorphisms involved in the study

Pathways	Genes	Polymorphisms	References
DNA repair	ERCC1	C354T (Asn118Asn)	7,10,11,14,16,17,19–22,41,44,46,51,67,69
		C8092A (Gln504Lys)	16,17,19–23,47,51
Detoxification	GSTP1	A313G (Ile105Val)	7,15,38,51,65,67
Transporter	MDR1	C3435T (Ile154Ile)	10,11,13,18,39
		G2677T/A (Ala893Ser/Thr)	10,13,18
DNA repair	XPD	A2251C (Lys751Gln)	7,11,12,21,22,35,41–49,51,69
		G934A (Asp312Asn)	7,11,12,22,43–45,48,49,51,69
DNA repair	XPG	G3310C (His1104Asp)	8,36,38
		T138C (His46His)	8,9,36,38
DNA repair	XRCC1	G1196A (Arg399Gln)	7,15,22,36–38,40,46,50–52
		C580T (Arg194Trp)	35–38,40,52
DNA repair	XRCC3	C18067T (Lys27Gln)	7,22,36,41,42,52
Metabolism	CDA	A79C (Thr241Met)	7,41,45,69

Genomic DNA of all subjects was isolated from a 5 mL peripheral blood sample using the FlexiGene DNA Kit (Qiagen, Hilden, Germany) and stored at −20C until use. Genotyping was conducted by the Sequenom MassARRAY system (Sequenom, San Diego, CA, USA).

### Publication search and inclusion criteria

We did a systematic literature search in PubMed database, ISI Web of Knowledge and Cochrane Library. The identified articles were reviewed carefully to find more relevant articles. The included articles were published before November 23^th^ 2015. Keywords for searching the related publications were platinum (platinum, cisplatin, carboplatin, oxaliplatin) and polymorphism (polymorphism, SNP, mutation, variation, single nucleotide polymorphism) and lung cancer.

The inclusion criteria of publications were as follows: (1) studies about platinum-based chemotherapy response; (2) Patients with NSCLC; (3) the data of genotypes in responders and nonresponders could be obtained. Studies were excluded by any one of the following conditions: (1) the data of genotypes could not be provided; (2) articles involved in patients received surgery or radiotherapy.

### Data extraction

All data were extracted independently by two investigators (JC and ZW) using the same data recording form, but they were blind to each other during the whole extracting process. The discrepancies of the extracted data were discussed and resolved with consensus. The following information were collected from each study: first author's name, publication year, ethnicity, country, sample size, polymorphisms, alleles of the investigated polymorphism, genotyping methods, disease stage, chemotherapy regimen, and the numbers of responders and non-responders in different genotypes.

### Statistical analysis

In our genotyping study, the chi-square and Student t tests were used to determine the differences in sex, age, smoking status and histology between responders and nonresponders. Unconditional logistic regression was performed to estimate the association of the polymorphisms with chemotherapy response by calculating odds ratios and their 95% confidence intervals with adjustments. The association study was analyzed in additive, dominant and recessive models. The *P* value was 2 sided, and *P* < 0.05 was considered statistically significant. The aforementioned statistical analyses were performed by PLINK 1.07 [28] and SPSS 18.0 (IBM, Armonk, NY, USA).

In the meta-analysis, the pooled odds ratio (OR) and associated 95% confidence interval (95% CI) were calculated by using the Z test. The genetic model was chosen by logistic regression [29]. The heterogeneity of publications in each meta-analysis was assessed by using Q statistic test, it with a significance level of *P* < 0.05. We selected the random-effect model to get the results with a wider CIs if *P* < 0.05. Otherwise, the fixed-effect model was used to calculate the pooled ORs and *P* values [30, 31]. To further evaluate the extent of heterogeneity between publications, I^2^ statistic test was also employed, its values of 25%, 50% and 75%were considered as low, moderate and high heterogeneity respectively [32]. The publication bias was examined by the inverted funnel plots, Begg's test [33]and Egger's test [34]. All calculations were conducted by Stata 12.0 (StataCorp LP, College Station, USA). The *P* value was 2 sided, and *P* < 0.05 was considered statistically significant.

## RESULTS

### Associations of the Polymorphisms with platinum-based chemotherapy response in genotyping study

1024 NSCLC patients were enrolled in our genotyping study and their clinical characteristics were summarized in Table 1. All of the patients received platinum-based chemotherapy at least two cycles. 237 of them showed good response while 787 had poor response to the treatment. 13 SNPs attempted to be genotyped by Sequenom's MassARRAY system, but 3 (XRCC1 C580T, CDA A79C, XRCC3 C18067T) of the SNPs were failed in primer design since primers of these 3 SNPs would form heterodimers with other primers. Additionally, 2 SNPs (MDR1 G2677T/A, XPD G934A) were not genotyped successfully in all samples, their genotyping results failed in Hardy-Weinberg equilibrium test. The results of associations between 8 SNPs and platinum-based chemotherapy were shown in Table 3 and Table S1. XRCC1 G1196A was significantly related to the platinum-based chemotherapy response. Patients with GA or GG genotypes were more sensitive to platinum-based chemotherapy. We also conducted subgroup analyses which samples selected by age (55 years old), sex, smoking status, histology or chemotherapy regimen. The results of subgroup analyses were summarized in Table 4. In patients with <55 years old, GSTP1 A313G and XPG G3310C were related to the chemotherapy response. In patients with ≥55 years old, ERCC1 C354T was associated with chemotherapy response. MDR1 C3435T, G2677T/A and XPD A2251C showed significant associations in patients of females. XRCC1 G1196A was related to drug response in smoking patients. In AC subgroup, ERCC1 C354T and XPG T138C were associated with platinum sensitivity. In patients with VP treatment, XRCC1 G1196A and MDR1 C3435T were correlated with platinum-based chemotherapy response.

**Table 3:** Association of XRCC1 G1196A with platinum-based chemotherapy response in our genotyping study

Gene	Polymorphisms	Genotype	Responders	Non-responders	Additive		Dominant		Recessive	
			*N*(%)	*N*(%)	OR(95%CI)	*P* value	OR(95%CI)	*P* value	OR(95%CI)	*P* value
XRCC1	G1196A	GG	104(43.9)	400(50.8)	0.80(0.63-1.02)	0.072	0.72(0.53-0.96)	0.028*	0.99(0.54-1.80)	0.968
		GA	110(46.4)	292(37.1)						
		AA	15(6.3)	47(6.0)						

* *P* < 0.05

**Table 4:** Stratification analyses of the associations of polymorphisms and platinum-based chemotherapy response in our genotyping study

Gene	Polymorphisms	Subgroups	Additive		Dominant		Recessive	
			OR(95%CI)	*P* value	OR(95%CI)	*P* value	OR(95%CI)	*P* value
GSTP1	rs1695	<55	2.03(1.18-3.46)	0.010*	2.36(1.29-4.28)	0.005*	1.49(0.32-6.88)	0.607
XPG	rs17655	<55	0.68(0.47-0.98)	0.038*	0.44(0.23-0.86)	0.016*	0.76(0.43-1.35)	0.348
ERCC1	rs11615	≥55	1.65(1.15-2.36)	0.006*	1.59(1.05-2.41)	0.028*	5.13(1.20-21.98)	0.028*
XPD	rs13181	Females	4.95(1.08-22.72)	0.040*	4.95(1.08-22.72)	0.040*		
MDR1	rs1045642	Females	0.67(0.46-0.99)	0.048*	0.55(0.30-1.00)	0.052	0.63(0.31-1.27)	0.194
MDR1	rs2032582	Females	1.43(0.98-2.09)	0.065	1.27(0.68-2.37)	0.453	2.44(1.17-5.05)	0.017*
XRCC1	rs25487	Smokers	0.74(0.53-1.02)	0.063	0.64(0.43-0.94)	0.024*	1.01(0.56-1.81)	0.945
ERCC1	rs11615	AC	1.85(1.17-2.92)	0.009*	2.05(1.18-3.58)	0.011*	2.73(0.79-9.40)	0.112
XPG	rs1047768	AC	0.53(0.36-0.77)	0.001*	0.52(0.32-0.86)	0.010*	0.30(0.13-0.65)	0.003*
XRCC1	rs25487	VP	0.62(041-1.15)	0.148	0.49(0.25-0.95)	0.035*	1.71(0.36-8.07)	0.496
MDR1	rs1045642	VP	0.62(0.39-0.99)	0.047*	0.70(0.35-1.42)	0.325	0.34(0.15-0.78)	0.011*

NSCLC: Non-Small Cell Lung Cancer; VP: Navelbine /Platinum; AC: Adenocarcinoma

* *P* < 0.05

### Results of meta-analysis

#### Characteristics of eligible studies

Overall 4014 studies were selected during the first step of systematic literature review about platinum and lung cancer. With further reviewed, there were 475 studies were involved in single nucleotide polymorphisms. After reviewing the abstracts, 32 reviews or meta-analyses and 306 irrelevant studies were excluded. After reading the full texts of the 137 articles which left for reviewed in next step, we found that 41 articles focused on prognosis or toxicity of platinum-based chemotherapy, 21 lacked enough information, 19 were *in vitro* studies, 9 were about small cell lung cancer, 7 involved in patients with surgery or radiotherapy, and 1 was duplicated publication. Finally, there were 39 publications and our genotyping study included in meta-analysis. The publications included 13 SNPs in 8 genes (Figure 1). The characteristics of these studies were summarized in Table 5. Funnel plot, Begg's test and Egger's test were used to estimate publication bias among the included studies. Visual inspection of the funnel plot of SNPs revealed a symmetrical inverted V shape (Figure S1).

**Table 5:** Characteristics of eligible publications considered in the meta-analysis

Authors	Year	Ethnicity(Country)	Number of patients	Disease stage	Chemotherapy regimans	Genotyping methods	Genes and polymorphisms	Reference number
Ryu et al.	2003	Asian (Korea)	109	IIIB-IV	cisplatin-based chemotherapy	SNaPShot assay	ERCC1 C354T,XPD A2251C,XPD G934A	44
Isla et al.	2004	Caucasian (Spain)	62	IIIB-IV	cisplatin-docetaxel	TaqMan	ERCC1 C354T,XPD A2251C,XPD G934A,MDR1 C3435T	11
Yuan et al.	2005	Asian (China)	151	IIIB-IV	platinum-based chemotherapy	PCR-RFLP	XPD A2251C, ERCC1 C8092A	47
Booten et al.	2006	Caucasian (United Kingdom)	89	III-IV	platinum-based chemotherapy	Direct sequencing	GSTP1 A313G	65
Yuan et al.	2006	Asian (China)	200	IIIB-IV	platinum-based chemotherapy	PCR-RFLP	XPD A2251C, XRCC1 C580T	35
Booton et al.	2006	Caucasian (United Kingdom)	89	III-IV	platinum-based chemotherapy	PCR-RFLP	XPD A2251C,XPD G934A	12
Pan et al.	2008	Asian (China)	69	IIIB-IV	cisplatin-vinorelbine	PCR-RFLP	MDR1 C3435T,MDR1 G2677T	18
Sun et al.	2008	Asian (China)	87	IV	platinum-based chemotherapy	3D polyacrylamide gel-based DNA microarray	XPG G3310C,XPG T138C,XRCC1 G1196A,XRCC1 C580T	38
Tibaldi et al.	2008	Caucasian (Italy)	65	IIIB-IV	cisplatin-gemcitabine	Taqman probe–based assays	ERCC1 C354T, XPD A2251C,XPD G934A,CDA A79C	69
Hong et al.	2009	Asian (China)	164	III-IV	cisplatin-vinorelbine	PCR-RFLP	XRCC1 G1196A,XRCC1 C580T	40
Kalikaki et al.	2009	Caucasian (Greece)	119	IIIA-IV	platinum-based chemotherapy	PCR-RFLP Direct sequencing	ERCC1 C354T,ERCC1 C8092A,XPD A2251C,XPD G934A,XRCC1 G1196A,GSTP1 A313G	51
Pan et al.	2009	Asian (China)	54	IIIB-IV	cisplatin-docetaxel	PCR-RFLP	MDR1 C3435T, MDR1 G2677T	13
Sun et al.	2009	Asian (China)	113	IIIA-IV	platinum-based chemotherapy	3-D polyacrylamide gel-based DNA microarray	GSTP1 A313G	38
Feng et al.	2009	Asian (China)	115	III-IV	platinum-based chemotherapy	Gelbased DNA microarray	XPG T138C	9
Chen et al.	2010	Asian (China)	95	IIIB-IV	cisplatin-based chemotherapy	ligase detection reactions	ERCC1 C354T,MDR1 C3435T,MDR1 G2677T	10
Li F et al.	2010	Asian (China)	115	IIIB-IV	platinum-based chemotherapy	3-D polyacrylamide gel-based DNA microarray	ERCC1 C354T,ERCC1 C8092A,XPD A2251C	21
Wang et al.	2010	Asian (China)	90	IIIB-IV	cisplatin-based chemotherapy	Direct sequencing	ERCC1 C354T,ERCC1 C8092A	16
Joerger et al.	2011	Caucasian (Netherlands)	137	IIIB-IV	platinum-gemcitabine	DNA sequencing	ERCC1 C354T,XPD A2251C,XPD G934A,XRCC1 G1196A,XRCC3 C18067T,GSTP1 A313G,CDA A79C	7
KimCurran et al.	2011	Asian (China)	300	IIIB-IV	cisplatin-based chemotherapy	RT-PCR	ERCC1 C8092A	23
Ludovini et al.	2011	Caucasian (Italy)	192	IIIB-IV	cisplatin-based chemotherapy	Taqman	ERCC1 C354T,XPD A2251C,CDA A79C,XRCC3 Thr241Met	41
Zhou et al.	2011	Asian (China)	111	IV	platinum-based chemotherapy	PCR-RFLP	GSTP1 A313G,XRCC1 G1196A	52
Xu et al.	2011	Asian (China)	130	IIIB-IV	platinum-based chemotherapy	PCR-RFLP	XRCC1 rs25487 G1196A,XRCC1 rs1799782 C580T,XRCC3 C18067T	39
Yan et al.	2011	Asian (China)	103	IIIB-IV	platinum-based chemotherapy	RT-PCR	MDR1 C3435T	15
Li D et al.	2012	Asian (China)	89	IIIA-IV	cisplatin-based chemotherapy	Gene sequencing analysis technique	ERCC1 C354T,XPD A2251C,XRCC1 G1196A	46
Krawczyk et al.	2012	Caucasian (Poland)	43	IIIB-IV	platinum-based chemotherapy	PCR-RFLP	ERCC1 C354T	14
Liao et al.	2012	Asian (China)	62	IIIB-IV	cisplatin-based chemotherapy	SNPstream UHT	ERCC1 C354T,ERCC1 C8092A,XPD A2251C,XPD G934A,XRCC1 G1196A,XRCC3 C18067T	22
Wu et al.	2012	Asian (China)	353	IIIA-IV	platinum-based chemotherapy	Sequenom MassARRAY	XPD A2251C,XPD G934A	49
Chen et al.	2012	Asian (China)	256	IIIB-IV	platinum-based chemotherapy	PCR-RFLP	XPD A2251C, XRCC3 C18067T	42
Hong et al.	2013	Asian (China)	135	IIIB-IV	platinum-based chemotherapy	TaqMan assays	ERCC1 C354T,ERCC1 C8092A	17
Huang et al.	2013	Asian (China)	187	IIIA-IV	platinum-based chemotherapy	MassARRAY	ERCC1 C354T,ERCC1 C8092A	19
Li X et al.	2013	Asian (China)	496	IIIA-IV	platinum-based chemotherapy	Sequenom MassARRAY	XPD A2251C,XPD G934A	48
Zhang et al.	2013	Asian (China)	451	IIIA-IV	platinum-based chemotherapy	TaqMan	XPG G3310C,XPG T138C	8
Lv et al.	2014	Asian (China)	91	IIIB-IV	cisplatin-based chemotherapy	TaqMan-MGB	ERCC1 C354T,GSTP1 A313G	67
Peng et al.	2014	Asian (China)	235	IIIA-IV	cisplatin-based chemotherapy	PCR-RFLP	XRCC1 G1196A	50
Zhang et al.	2014	Asian (China)	375	IIIA-IV	platinum-based chemotherapy	Sequenom MassARRAY	XRCC1 G1196A,XRCC1 C580T	37
Zhao et al.	2014	Asian (China)	162	IIIA-IV	platinum-based chemotherapy	MassARRAY	ERCC1 C354T, ERCC1 C8092A	20
Zhou et al.	2014	Asian (China)	93	IIIB-IV	cisplatin-based chemotherapy	PCR-RFLP	XPD A2251C,XPD G934A,CDA A79C	45
Jin et al.	2014	Asain(China)	378	I-IV	cisplatin-based chemotherapy	PCR-RFLP	XPG G3310C,XPG T138C,XRCC1 G1196A,XRCC1 C580T,XRCC3 C18067T	36
Li P et al.	2015	Asian (China)	142	IIIB-IV	cisplatin-Vinorelbine	PCR-RFLP	XPD A2251C,XPD G934A	43

#### Pooled analysis of chemotherapy response

**Figure 2:**
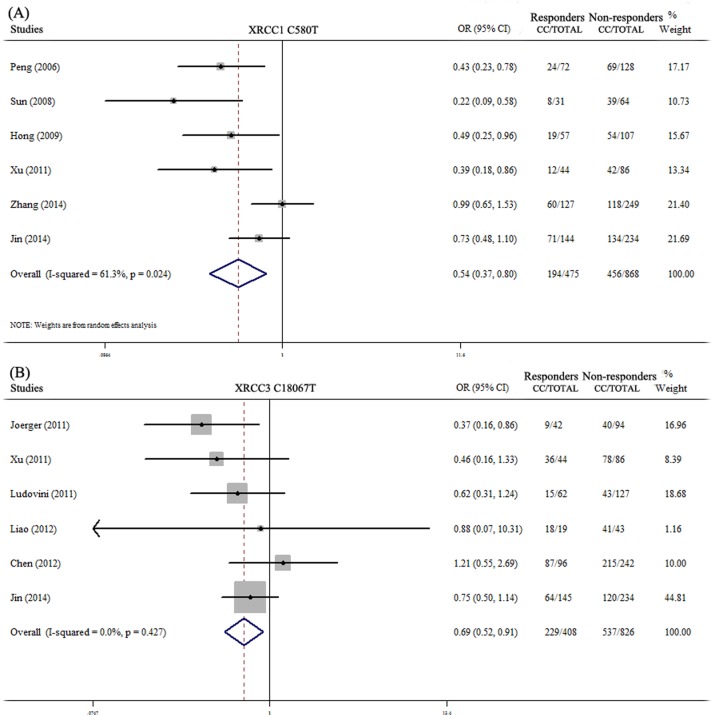
Meta-analysis of associations of XRCC1 C580T and XRCC3 C18067T with platinum-based chemotherapy response in NSCLC patients

The pooled estimate results of the 13 SNPs were shown in Figure 2 and Figure S2. 6 studies examined the association of XRCC1 C580T with platinum sensitivity in NSCLC patients [35–40]. It included 1343 subjects which 650 carried CC genotype and 693 with CT+TT genotype. We chose random-effect model since there was heterogeneity across the studies (*P* = 0.026, I^2^ = 61.3%). Pooled data showed there was significant relationship between XRCC1 C580T and drug response (OR = 0.54, 95%CI: 0.37-0.80, *P* = 0.002) (Figure 2A). Patients of XRCC1 C580T CT and TT carriers had better response than CC carriers. Meta-analysis of XRCC3 C18067T included 6 studies [7, 22, 36, 39, 41, 42]. There was no heterogeneity across these studies (*P* = 0.427, I^2^ = 0%), thus we chose fixed-effect model. Pooled data contained 1234 NSCLC patients, 408 were responders and 826 were non-responders. The pooled estimated result showed that this polymorphism was significantly correlated with chemotherapy response (OR = 0.69, 95%CI: 0.52-0.91, *P* = 0.009) (Figure 2B). XRCC3 C18067T CT and TT genotypes carriers showed better drug response. However, no significant associations were found between the other polymorphisms and chemotherapy response (Figure S2).

#### Stratified analyses of chemotherapy response

**Figure 3:**
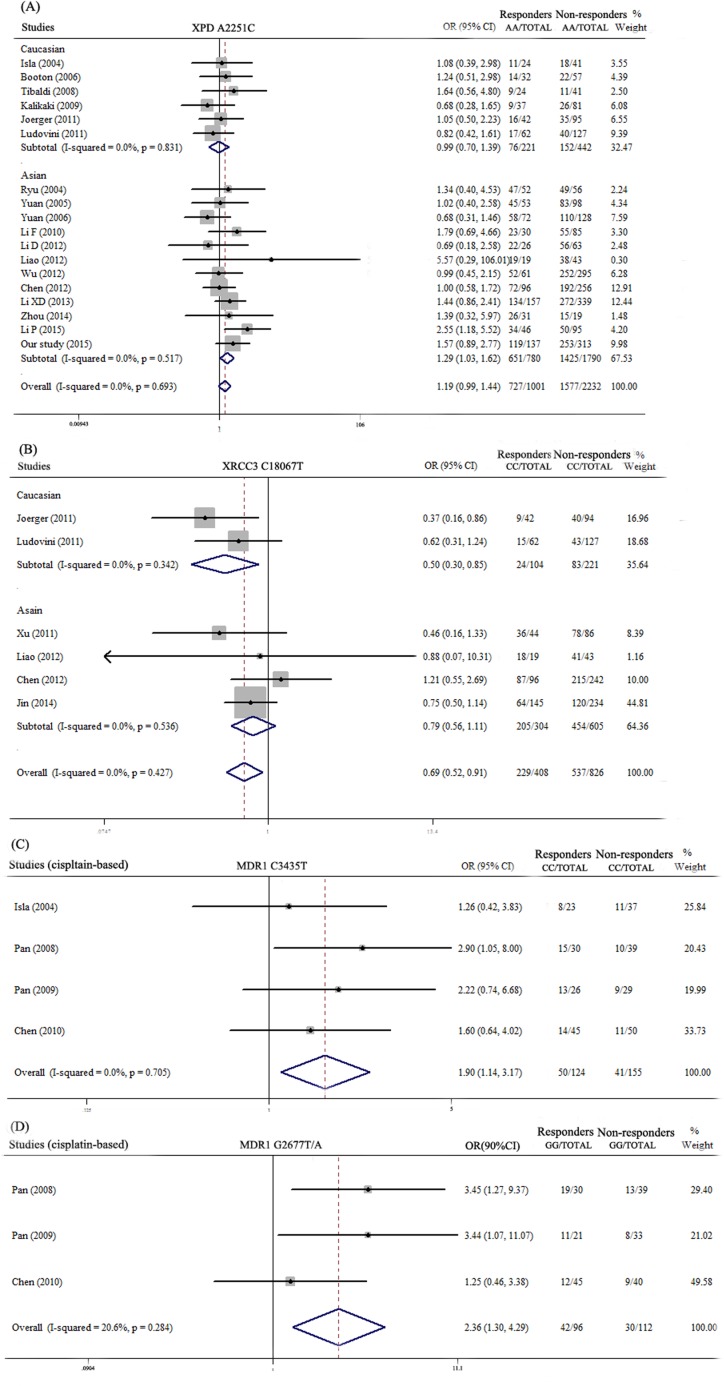
Meta-analysis of association polymorphisms of chemotherapy response in different subgroups

**Figure 4:**
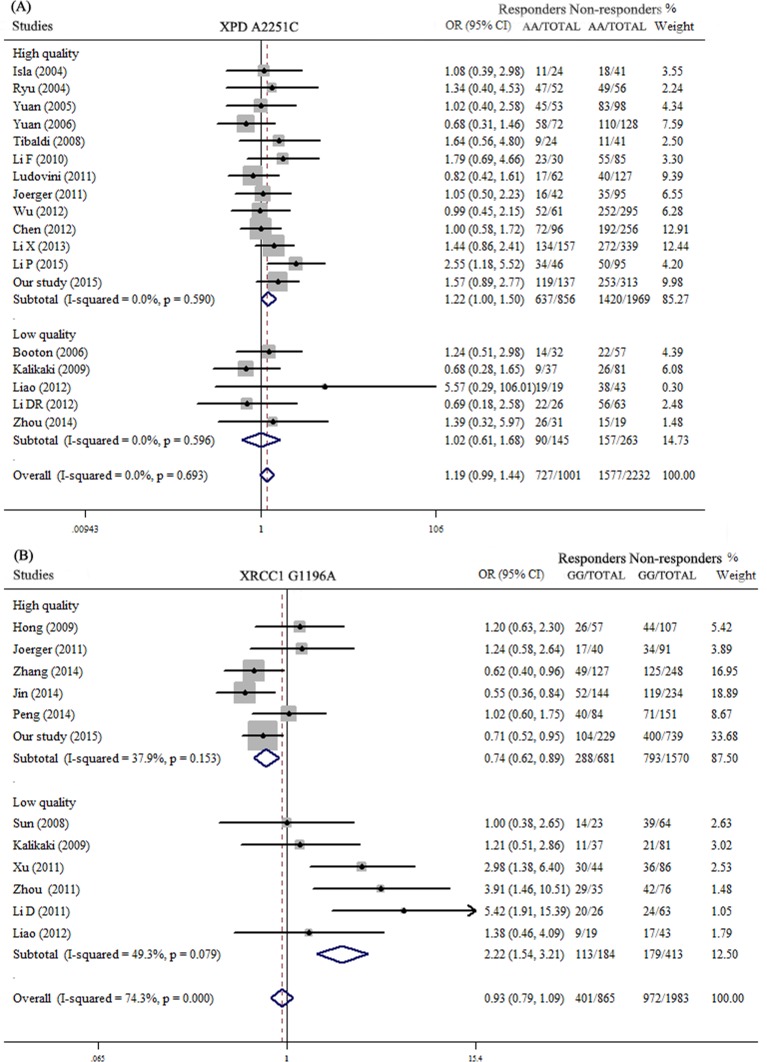
Meta-analysis of association polymorphisms of chemotherapy response after quality assessment of studies

Since moderate or high heterogeneity was showed in pooled analysis of several SNPs, and for considering that ethnic differences and different chemotherapy regimen may contribute to the drug response, we conducted stratified analyses of Asian, Caucasian and cisplatin-based chemotherapy. It was interesting to found that XPD A2251C was significantly correlated with platinum response in Asian population by pooling 12 studies [7, 21, 22, 35, 42–49] included 2570 subjects (OR = 1.29, 95%CI: 1.03-1.62, *P* = 0.026) (Figure 3A). Patients of Asians with XPD A2251C AA genotype could be more sensitive to platinum-based chemotherapy. XRCC3 C18067T was significantly related to drug response in Caucasian population (OR = 0.50, 95%CI: 0.30-0.85, *P* = 0.011) (Figure 3B). Patients of Caucasians with CT and TT genotypes of this polymorphism had better drug response. By only pooling the investigations that patients received cisplatin-based chemotherapy, MDR1 C3435T and MDR1 G2677T/A were significantly correlated to chemotherapy response (OR = 1.90, 95%CI: 1.14-3.17, *P* = 0.013; OR = 2.36, 95%CI: 1.30-4.29, *P =* 0.005, respectively) (Figure 3C and 3D). Patients carrying CC genotype of MDR1 C3435T or GG genotype of MDR1 G2677T/A had better chemotherapy response. There were 11 publications studied association of XRCC1 G1196A [7, 22, 36–40, 46, 50–52], but high heterogeneity (*P*<0.001, I^2^ = 74.3%) and publication bias exist among the studies (Begg's test *P* = 0.024; Egger's test *P =* 0.002). Moreover, it was hardy to determine about the association of XPD A2251C with chemotherapy response for its 95%CI was 0.99-1.44. Thus, we made quality assessment about the publications by using quality scoring criteria from Wu's study [26], but it was modified by considering high (or low) quality if the score >12 (or ≤12) in our analyses. We did quality stratified analyses in SNPs which reported in more than 10 studies. The results showed that XPD A2251C was more likely related to drug response in pooled analysis of high quality publications which included 2625 patients (OR = 1.22, 95%CI: 1.00-1.50, *P* = 0.05) (Figure 4A), and XRCC1 G1196A was associated with chemotherapy response in high quality publications with low heterogeneity. (OR = 0.74, 95%CI: 0.62-0.89, *P* = 0.001) (Figure 4B).

#### Quality assessment of meta-analysis results

The reliability of our meta-analysis results were assessed by the criteria described in Table S2. Score was determined by the following 4 factors: number of included publications, sample size of patients, heterogeneity and publication bias. Total score ranged from 2 to 12. Meta-analysis results were considered low or medium or high reliability if the score were 2-6 or 7-9 or 10-12. The quality assessment results were described in Table 6. We were exciting to notice that high reliability showed about XRCC1 G1196A pooled analysis result in high quality studies, while the total pooled analysis result was in low reliability. Moreover, our genotyping study result of XRCC1 G1196A was consistent with the high reliability result.

**Table 6:** Quality assessment of meta-analysis results

Genes	SNPs	Total pooled analysis reliability (score)	Subgroup analysis reliability (score)				
			High quality studies	Low quality studies	Asian	Caucasian	Cisplatin
ERCC1	C354T	High (10)	High (10)	Medium (7)	Medium (9)	Medium (7)	Medium (8)
	C8092A	High (11)	Medium (9)	Medium (8)	Medium (9)		Medium (8)
GSTP1	A313G	Medium (8)			Low (6)	Medium (8)	
MDR1	C3435T	Medium (9)			Medium (8)		Medium (8)
	G2677T/A	Medium (7)					Medium (8)
XPD	A2251C	High (12)	High (12)	Medium (8)	High (12)	Medium (9)	Medium (9)
	G934A	High (12)	Medium (9)	Medium (8)	Medium (9)	Medium (8)	Medium (7)
XPG	G3310C	Medium (9)					
	T138C	Medium (7)					
XRCC1	G1196A	Low (6)	High (10)	Medium (8)	Medium (9)	Medium (8)	
	C580T	Medium (8)					
CDA	A79C	Medium (7)				Medium (7)	Low (6)
XRCC3	C18067T	High (10)			Medium (8)	Medium (8)	

## DISCUSSION

In this study, we investigated the associations of widely studied SNPs (13 polymorphisms in 8 genes) with platinum-based chemotherapy response in NSCLC patients. We also conducted a comprehensive meta-analysis of these SNPs. Our results showed that XRCC1 G1196A/C580T, and XRCC3 C18067T were significantly correlated with platinum-based chemotherapy. XPD A2251C, MDR1 C3435T and MDR1 G2677T/A, GSTP1 A313G, XPG G3310C, ERCC1 C354T were correlated to platinum-based chemotherapy response in different subgroups.

Platinum-based chemotherapy was widely used for treatment of advanced NSCLC, but pharmacogenomic differences between individuals may affect drug response. In the last several decades, gene polymorphisms were revealed to play an important role in chemotherapy response [24]. Therefore, most studies focused on the polymorphisms of genes involved in DNA repair pathway, transporters, metabolism and detoxification. In this study, we selected 9 SNPs in DNA repair pathway genes, 2 SNPs of transporter genes and 2 from metabolism and detoxification genes. They were extensively studied by researchers but results were not consistent. We used a larger sample size (n = 1024) to detect relationships between these SNPs and platinum-based chemotherapy response in NSCLC. Moreover, we conducted a meta-analysis to verify the results.

DNA repair was one of the classical platinum resistance mechanism [53]. Polymorphisms in genes of DNA repair pathway could affect DNA repair capacity and alter sensitivity to platinum-based chemotherapy. In this study, XRCC1 G1196A was significantly correlated to platinum-based chemotherapy response both in our genotyping study and meta-analysis. XRCC1 C580T and XRCC3 C18067T were also related to the drug response from the meta-analysis. XRCC1 and XRCC3 were both DNA base excision repair (NER) genes. XRCC1 G1196A/C580T and XRCC3 C18067T were all non-synonymous SNPs. They could directly contribute to gene expression and activity. Increased NER capacity would lead to decrease sensitivity of patients to chemotherapy [54]. A recent meta-analysis conducted by Gao et.al [55] about XRCC1 G1196A and C580T showed consistent result with our findings about XRCC1 C580T, but no significantly association showed about XRCC1 G1196A in their study. We carefully compared the included studies in the two meta-analyses. Most of studies included in their meta-analysis were small sample size studies, and they also included the studies with patients received radiotherapy which we excluded [56, 57]. For DNA repair gene polymorphisms, additionally, we also found that XRCC3 C18067T showed contribution to drug response in Caucasians and XPD A2251C was related to the response in Asian population. Allele frequency usually different between races, and racial differences were important factors for drug response [58, 59]. We did not find any statistical evidence for associations between the other SNPs (ERCC1 C354T/C8092A, XPD G934A and XPG G3310C/T138C) in DNA repair pathway and drug response in the meta-analysis. In our genotyping study, these polymorphisms also showed no significant correlations to platinum-based chemotherapy response in the overall analysis.

Transporters involved in drug resistance by decrease uptake or efflux of the drugs by the proteins known as ATP binding cassette transporters [60]. MDR1 also named ABCB1, it encodes P-glycoprotein which is an ATP-dependent drug efflux. It is responsible for decreased drug accumulation and often mediates the development of resistance to anticancer drug [61–63]. We found that both MDR1 C3435T and G2677T/A were associated with cisplatin-based chemotherapy response from the results of meta-analysis. However, the numbers of subjects pooled for these two SNPs in cisplatin-based chemotherapy subgroup were small. We considered that these associations maybe need further investigations. Additionally, there were several findings in our genotyping study about the SNPs. Both MDR1 C3535T and G2766T/A were associated with platinum-based chemotherapy response in females, and MDR1 C3435T also related to platinum response in AC patients.

GSPT1 and CDA were metabolism and detoxification pathway genes. GSTP1 was a member of GST family. It mediates formation of platinum-glutathione adduct [64] which decreased chemotherapy sensitivity. 7 studies were included in GSTP1 A313G pool analysis [51, 52, 65–67], but no significant correlation between this polymorphism and chemotherapy response. However, we found GSTP1 A313G related to platinum-based chemotherapy response of patients with <55 year old in our genotyping study. CDA is an enzyme that metabolic inactivation of gemcitabine [68]. The patients in 4 studies of CDA A79C were received platinum-gemcitabine treatment [7, 41, 45, 69], but no associations were found in the meat-analysis of CDA A79C.

We conducted this study to get comprehensive conclusions about SNPs contributed to platinum-based chemotherapy response in NSCLC patients, but there were several possible limitations. It was unfortunately about the genotyping of XRCC1 C580T and XRCC3 C18067T failed in our samples, thus meta-analyses of these two SNPs were calculated without the results from our genotyping study. In meta-analyses of ERCC1 and MDR1, we did not include the data from the studies by Krawczyk et al. [70] and Du et al. [71] because they classified SD patients as responders. Our meta-analysis mainly used unadjusted estimates because not all publications presented their adjusted estimates. Finally, we attempted to conduct meta-analysis in each subgroup, but the other publications did not conduct subgroup analysis, or when they did but not in the same way. Thus, we cannot do pool analyses in the subgroups.

It was our first attempt to evaluate the quality of meta-analysis results. Only ERCC1 C3435T/C8092A, XPD A2251C/G934A, XRCC1 G1196A and XRCC3 C18067T showed high reliability. It means the other SNPs may need more investigations, though most of them showed medium reliability. In conclusion, contributions of XRCC1 G1196A/C580T and XRCC3 C18067T to drug response were further confirmed by our large sample size genotyping study and comprehensive meta-analysis. It is meaningful for personalized platinum-based treatment of NSCLC.

## SUPPLEMENTARY MATERIALS


