# Supplementary material for: Pharmacogenomics of platinum-based chemotherapy response in NSCLC: a genotyping study and a pooled analysis
Source: Oncotarget. 2016 May 29;7(34):55741–56. doi: 10.18632/oncotarget.9688 (PMC5342450; doi:10.18632/oncotarget.9688)
Supplement: Supplementary file 1 [file oncotarget-07-55741-s001.pdf]

# Pharmacogenomics of platinum-based chemotherapy response in NSCLC: a genotyping study and a pooled analysis

**Supplementary Material**

Table S1 Associations of polymorphisms in ERCC1, GSTP1, MDR1, XPD and XPG with platinum-based chemotherapy response in our genotyping study

| Gene | Polymorphisms | Genotype | Responders | Non-responders | Additive | | Dominant | | Recessive | |
|---|---|---|---|---|---|---|---|---|---|---|
| | | | N(%) | N(%) | OR(95%CI) | P value | OR(95%CI) | P value | OR(95%CI) | P value |
| ERCC1 | rs11615 | CC | 129(58.4) | 342(49.6) | 1.30(0.99-1.69) | 0.055 | 1.31(0.94-1.81) | 0.105 | 1.78(0.85-3.71) | 0.124 |
| | | CT | 69(31.2) | 224(32.5) | | | | | | |
| | | TT | 9(4.1) | 46(6.7) | | | | | | |
| | rs3212986 | CC | 102(43.0) | 335(42.6) | 0.96(0.78-1.19) | 0.740 | 0.97(0.72-1.30) | 0.826 | 0.92(0.60-1.42) | 0.723 |
| | | CA | 97(40.9) | 322(40.9) | | | | | | |
| | | AA | 32(13.5) | 99(12.6) | | | | | | |
| GSTP1 | rs1695 | AA | 162(68.4) | 483(61.4) | 1.25(0.95-1.65) | 0.110 | 1.32(0.96-1.83) | 0.090 | 1.22(0.55-2.69) | 0.622 |
| | | AG | 59(24.9) | 231(29.4) | | | | | | |
| | | GG | 8(3.4) | 32(4.1) | | | | | | |
| MDR1 | rs1045642 | CC | 81(34.2) | 301(38.2) | 0.88(0.72-1.09) | 0.247 | 0.82(0.60-1.12) | 0.213 | 0.89(0.60-1.32) | 0.565 |

| | | | | | | | | | | |
|---|---|---|---|---|---|---|---|---|---|---|
| | | CT | 107(45.1) | 330(41.9) | | | | | | |
| | | TT | 40(16.9) | 121(15.4) | | | | | | |
| XPD | rs13181 | AA | 119(86.9) | 253(80.8) | 1.29(0.77-2.15) | 0.334 | 1.53(0.86-2.72) | 0.150 | 0.36(0.07-1.88) | 0.227 |
| | | AC | 15(10.9) | 57(18.2) | | | | | | |
| | | CC | 3(2.2) | 3(1.0) | | | | | | |
| XPG | rs17655 | GG | 48(20.3) | 199(25.3) | 1.12(0.91-1.38) | 0.282 | 1.02(0.72-1.44) | 0.907 | 1.35(0.94-1.94) | 0.100 |
| | | GC | 118(49.8) | 357(45.4) | | | | | | |
| | | CC | 58(24.5) | 190(24.1) | | | | | | |
| | rs1047768 | TT | 117(49.4) | 395(50.2) | 0.90(0.71-1.13) | 0.365 | 0.93(0.69-1.24) | 0.610 | 0.73(0.43-1.23) | 0.232 |
| | | TC | 95(40.1) | 321(40.8) | | | | | | |
| | | CC | 22(9.3) | 53(6.7) | | | | | | |

Table S2 Scale for quality assessment of meta-analysis results

| Criteria | Item | Score |
| --- | --- | --- |
| Number of studies | ≥10 | 3 |
| | ＞5 but ＜10 | 2 |
| | ≤5 | 1 |
| Sample size of patients | ≥2000 | 3 |
| | ＞1000 but ＜2000 | 2 |
| | ≤1000 | 1 |
| Heterogeneity | None ($I^2$＜25%) | 3 |
| | Low ($25\% \leq I^2 < 50\%$) | 2 |
| | Medium ($50\% \leq I^2 < 75\%$) | 1 |
| | High ($I^2 \geq 75\%$) | 0 |
| Publication bias | No | 3 |
| | Yes | 0 |

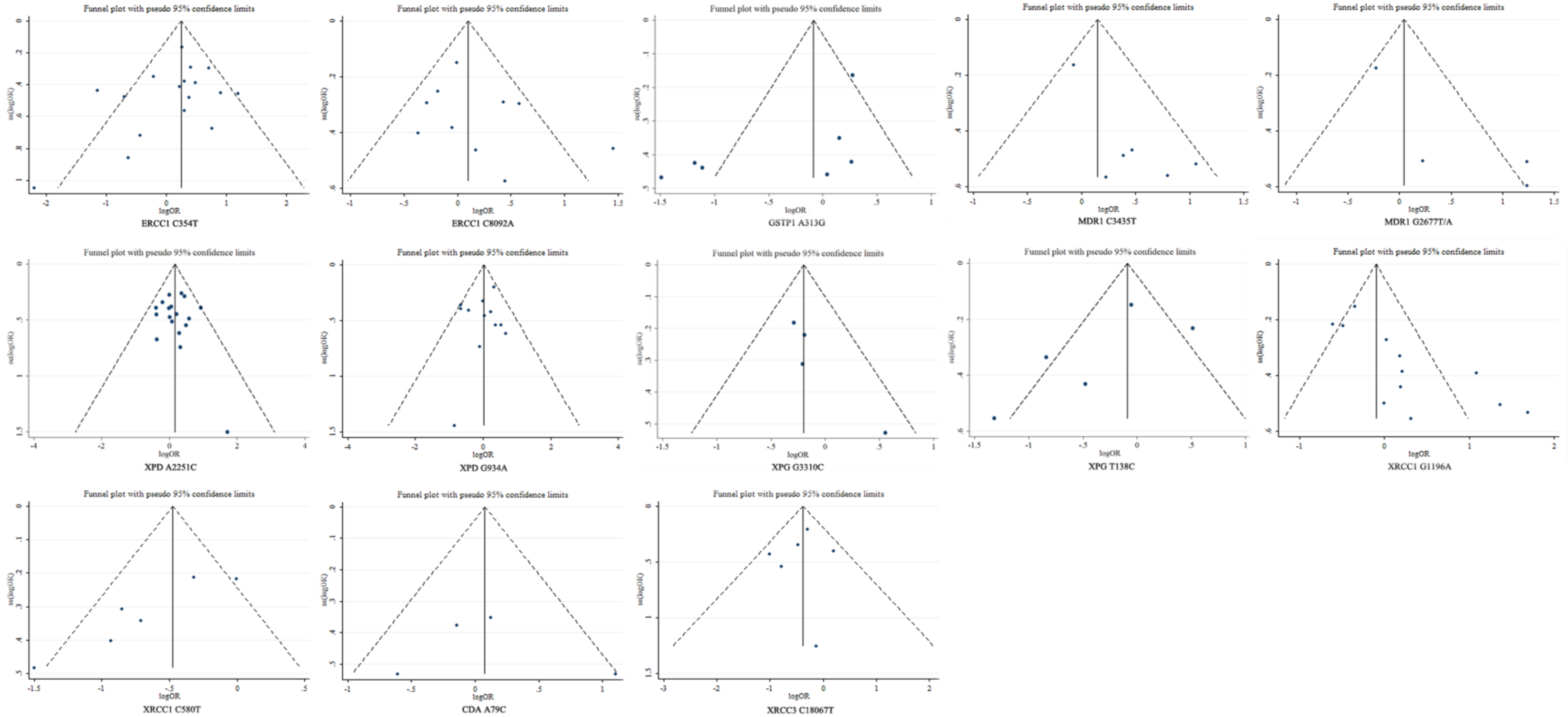


**Figure S1** Funnel plots of SE (log [OR]) by the logOR in meta-analysis. SE (log [OR]) is standard error of log [OR]. Each dot represents one article.

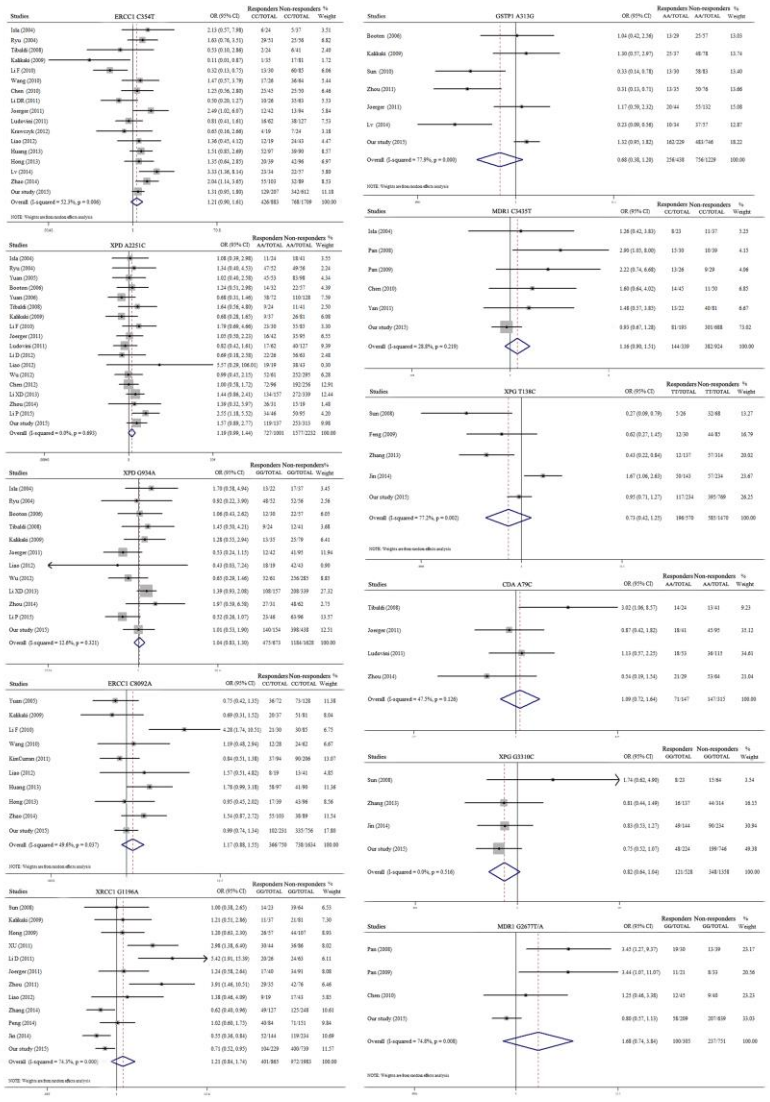

**Figure S2** Meta-analysis of polymorphisms that no significantly associations with platinum-based chemotherapy response.
